# Development and validation of a larval bioassay and selection protocol for insecticide resistance in *Drosophila suzukii*

**DOI:** 10.1371/journal.pone.0270747

**Published:** 2022-06-29

**Authors:** Brian E. Gress, Frank G. Zalom

**Affiliations:** Department of Entomology & Nematology, University of California Davis, Davis, California, United States of America; University of Thessaly School of Agricultural Sciences, GREECE

## Abstract

The rapid invasion of *Drosophila suzukii* (Matsumura) throughout Europe and the Americas has led to an increased reliance on calendar-based broad-spectrum insecticide programs among berry and cherry growers. Relatively few active ingredients (AIs) are currently available for effective *D*. *suzukii* management, and studies from multiple growing regions indicate that susceptibility to at least some of these materials is declining. Greater effort is needed to understand the status of susceptibility across field populations and the potential for increased resistance to develop, as well as the possible fitness costs incurred by resistant individuals. However, current bioassay protocols used for resistance monitoring and selection studies (i.e. resistance risk assessments) are labor-intensive and costly, making large-scale studies difficult to conduct. Here, we first present a novel bioassay protocol using larvae that requires little effort or cost to implement beyond what is needed for basic *D*. *suzukii* laboratory colony maintenance. We then perform dose-response bioassays using this protocol to identify larval lethal concentrations for three commonly used insecticides (malathion, spinosad and zeta-cypermethrin) in a susceptible population. Finally, resistance risk assessments were conducted using a population of *D*. *suzukii* from commercial caneberry fields near Watsonville, CA. We find that five generations of larval selection with a discriminating dose is sufficient to significantly increase both larval (malathion and spinosad) and adult (spinosad) resistance to the target AIs. This approach provides a simple, cost-effective tool for assaying susceptibility of *D*. *suzukii* populations to insecticides and for selecting resistant insect lines for resistance management research.

## Introduction

*Drosophila suzukii* (Matsumura), commonly known as the spotted-wing drosophila, is an invasive pest of small fruit and berry production native to Southeast Asia. Starting in 2008, this species underwent a rapid geographic range expansion, spreading across North America, South America, and Europe [1–3]. Following its arrival to the USA, growers in California, Oregon, and Washington reported yield losses of up to 50% in raspberries and blackberries, 40% in blueberries, and 33% in cherries [4]. Unlike other *Drosophila* species that typically breed on overripe and rotting fruit, *D*. *suzukii* females readily lay eggs into firm, still-ripening fruit using specialized serrated ovipositors [5]. After hatching, larvae continue to feed and develop within the fruit, causing rapid decay and increased susceptibility to subsequent infestation by other drosophilids.

Efforts to control *D*. *suzukii* and prevent infestation have led to a drastic increase in the use of broad-spectrum insecticide applications in susceptible host crops [6]. Specifically, three main classes of insecticide–organophosphates, pyrethroids, and spinosyns–have shown high efficacy and now form the basis of most conventional *D*. *suzukii* management programs [7–10]. Carbamates and some diamides have also shown moderate efficacy against *D*. *suzukii*, but the use of these materials is more limited throughout North America and Europe [8, 10]. The continued reliance on chemical sprays to control this pest, combined with its high fecundity and rapid development time, have elicited concerns that insecticide resistance could quickly develop. For organic growers, the risk of resistance is even greater. Currently, spinosad (a spinosyn) is the only insecticide approved by the Organic Materials Review Institute with high efficacy against *D*. *suzukii*, meaning that growers must rotate spinosad sprays with low-efficacy materials such as pyrethrins and azadirachtin in order to comply with label requirements [7, 8]. Consequently, *D*. *suzukii* in organic fields are primarily challenged by only one class of insecticide further increasing the risk of resistance. Indeed, recent work by Gress and Zalom [11] reported low to moderate levels of spinosad resistance in *D*. *suzukii* collected from commercial caneberry fields near Watsonville, CA. Specifically, this study utilized dose-response analysis of adult *D*. *suzukii* to quantify the degree of resistance and found that Watsonville females exhibited LC_50_s 5–8 times higher than females from a second, untreated location in California. This finding demonstrates an immediate need to expand our understanding of resistance evolution in *D*. *suzukii* and to develop novel tools that aid in the early detection and assessment of resistant populations so that appropriate management actions can be implemented.

When decreased susceptibility to an insecticide is first detected in a pest field population, laboratory selection studies, often referred to as resistance risk assessments, represent a critical first step towards understanding the potential for resistance to develop [12]. These studies are performed by exposing large numbers of individuals from the suspect field population to moderately lethal concentrations of insecticide for multiple generations such that the most susceptible individuals are eliminated and the most tolerant survive. Dose-response analyses are performed both before and after implementing the selection protocol, and the change in LC_50_ is used to quantify the response to selection and better understand the potential for resistance to develop [12, 13]. This approach, however, is not without its limitations. For example, because laboratory colonies contain limited genetic variation relative to large field populations, these studies can understate the evolutionary potential for resistance to develop (e.g. through novel genetic combinations or mutations) in a field setting [12]. Conversely, selection imposed in laboratory settings may be more intense and consistent than in the field, leading to possible overestimation of resistance development. Nevertheless, an increase in resistance due to laboratory selection provides unambiguous evidence that further loss of susceptibility in the field is possible [12]. Additionally, if laboratory selection is successful and resistant colonies are generated, these strains can assist researchers in the development of novel tools for detecting, monitoring, and managing resistance in the field. For example, resistant colonies can be used to identify the molecular mechanism(s) that produces the resistant phenotype, and these markers can, in some instances, be used to track spatiotemporal patterns of resistance development in the field [14]. Laboratory-generated resistant colonies can also be used to assess whether insecticide resistance in a particular population is associated with fitness costs (i.e. reproductive or survival impairments), and this information can be used to develop programs to manage resistance in the field. The presence of such costs under benign conditions would suggest that efforts to temporarily eliminate the target insecticide from spray programs could successfully maintain susceptibility in the field, at least in the short-term [15].

After decreased spinosad susceptibility in the Watsonville, CA population was identified, Gress and Zalom [11] performed a resistance risk assessment by continuing to expose adults from this population to a discriminating dose of spinosad using a standard glass vial bioassay protocol developed for *D*. *suzukii* [16]. After implementing this technique for 5 generations, Gress and Zalom [11] found that LC_50_ values increased by 86% for males and 49% for females. This selection protocol, however, is highly labor intensive and costly, making it impractical for long-term selection programs which are needed to generate and maintain resistant lines. Additionally, this method requires that researchers kill off much of their adult population each generation, increasing the risk that selection lines will be lost in the process. Here, we describe a simple larval bioassay that overcomes these challenges and requires little effort or cost beyond what is already needed for basic laboratory stock maintenance. We then perform a series of larval dose-response bioassays to calibrate the selection protocol and identify baseline susceptibility for three commonly used insecticides. Finally, we implement the larval bioassay protocol to perform resistance risk assessments using the same Watsonville, CA field strain previously tested by Gress and Zalom [11]. Changes in the susceptibility at both the larval and adult life stages were quantified, and results from this study are compared to those obtained with the original adult selection method.

## Materials and methods

### Statement of ethics

Insect collection clearance was obtained from the California Department of Fish and Wildlife (Scientific Collecting Permit no. SC-13698).

### Larval bioassay protocol for resistance selection

Here we explain the general larval bioassay protocol used throughout the paper. First, groups of n = 20 mated females (4–12 days post-eclosion) were transferred into plastic 6 oz *Drosophila* stock bottles (Fisher Scientific, Inc., Portsmouth, NH, USA) containing fresh Bloomington standard *Drosophila* cornmeal diet. Females were allowed to lay eggs for four days before being transferred into new bottles with fresh diet. Once females were removed, 400 μL of insecticide solution was pipetted onto the surface of the diet, and each bottle was shaken side-to-side to distribute the solution. This ensured that all larvae were uniformly exposed to the insecticide, both through external contact and internally while feeding on the treated diet. Control bottles were treated with either ddH_2_O or acetone, depending on the insecticide treatment (see below), but otherwise were handled using the same protocol as experimental bottles. All bottles were then re-plugged with a cotton stopper and stored in a climate-controlled walk-in chamber at 22–23°C and 14–10 light-dark cycle. Because the first larvae typically crawl out of the diet to pupate after 6 days under these conditions (pers observ), treatment timing is critical to ensure that all larvae are exposed. Bottles were monitored for up to 18 days following treatment for the emergence of adult *D*. *suzukii*, and all newly enclosed adults were transferred to fresh bottles and counted.

### Identifying larval lethal concentrations of commonly used insecticides

To quantify baseline susceptibility of *D*. *suzukii* larvae, we used the larval bioassay protocol to perform dose-response analyses for three insecticide AIs, each representing a different insecticide class, commonly used against this pest. This work was conducted using a susceptible laboratory strain of *D*. *suzukii* originally collected from USDA Wolfskill Germplasm Repository in Winters, CA (henceforth “WOLF”) in fall of 2017. Wolfskill is an experimental, mixed-fruit orchard, and crops at this location receive no insecticide treatment. This strain of *D*. *suzukii* was previously used as a susceptible control by Gress and Zalom [11] when quantifying resistance in the Watsonville population.

The three formulated insecticides used in this study were malathion (Malathion Insect Spray Concentrate, Spectrum Group, St. Louis, MO, USA), spinosad (Entrust® 24SC, Dow AgroSciences LLC, Indianapolis, IN, USA), and zeta-cypermethrin (MustangMaxx ^TM^ 0.8 EC, FMC Corporation, Philadelphia, PA, USA). Serial dilutions were performed for each product using either ddH_2_O (for spinosad) or acetone (for malathion and zeta-cypermethrin) to obtain a range of concentrations (spinosad: 0–10 parts per million (ppm), malathion: 0–40 ppm, and zeta-cypermethrin: 0–25 ppm). In total, 5–8 concentrations were used per insecticide, and each concentration was replicated across 2–5 bottles.

### Larval selection and susceptibility

Approximately n = 250 *D*. *suzukii* were live-trapped from four commercial caneberry fields near Watsonville, CA on October 23, 2018 using plastic McPhail traps (Great Lakes IPM, Inc., Vestaburg, MI, USA) baited with a mixture of approximately 7 g yeast, 113 g sugar, and 355 ml water. Traps were modified by fitting a mesh barrier such that *D*. *suzukii* adults could enter the trap unobstructed but were prevented from reaching the liquid lure. Traps were transported back to a laboratory facility on the University of California, Davis campus where all flies were transferred into 6 oz Drosophila bottles containing standard cornmeal diet, with approximately 30 flies per bottle. Every fourth day, Watsonville *D*. *suzukii* (henceforth “WAT”) were turned over into new bottles with fresh diet, and all progeny were reared to adulthood under standard laboratory conditions (described above). Starting in the F3 and F4 generations, we generated two selection lines: a spinosad selection line (WAT-S) and a malathion selection line (WAT-M). Each generation, larvae were treated with a discriminating dose of insecticide (approximately the LC_90x2_ for susceptible larvae; 10 ppm spinosad for WAT-S and 40 ppm malathion for WAT-M) following the larval selection protocol outlined above. All adult flies that emerged from treated bottles (i.e. “survivors”) were collected and transferred to fresh diet to produce the next generation. The number of adults to emerge from each insecticide-treated bottle was counted during the first generation of selection (WAT: n = 10 bottles/insecticide) and again after five generations (WAT-S_5_: n = 10 bottles; WAT-M_5_: n = 10 bottles) to measure change in larval susceptibility. Survival in the malathion selection line was again assessed after approximately 20 generations of selection (WAT-M_20_: n = 12 bottles). At each time point, bottles treated with either ddH_2_O (n = 3) or acetone (n = 3) only were included to allow estimation of baseline eclosion in the absence of insecticide. Additionally, WOLF bottles (n = 3/AI/timepoint) were treated with insecticide to serve as a susceptible point of comparison and ensure that the insecticide treatment was effective.

### Impact of larval selection on adult susceptibility

To assess the impact of larval selection on adult susceptibility, we used pre-selection F1 and F2 WAT (n = 685 per sex) and post-selection WAT-S_5_ (n = 295 per sex) adults to perform spinosad dose-response analysis, with concentrations ranging from 0–1000 ppm. Prior to testing WAT-S_5_ adults, insecticide treatment was removed for one generation to expand the size of the colony. Adults from the WOLF population were also tested concurrently with WAT and WAT-S_5_ and served as a susceptible point of comparison. During the pre-selection phase of bioassays, n = 275 WOLF adults (henceforth “WOLF-1”) of each sex were bioassayed, and during the post-selection phase n = 295 per sex (“WOLF-2”) were tested.

Adult bioassays were performed using the protocol developed by Van Timmeren et al [16] and used by Gress & Zalom [11] in which 3–8-day old laboratory-reared flies were exposed to insecticide residues within glass scintillation vials. In brief, 1 mL of insecticide solution was added to each 20-mL glass scintillation vial, and vials were then capped and shaken for five seconds before pouring out the excess liquid. To prevent the remaining insecticide solution from settling at the bottom, vials were inverted and rolled every 60 minutes for four hours during the drying process. The next morning, n = 5 male and n = 5 female *D*. *suzukii* were gently aspirated into each vial and left for 8 hrs. Mortality assessments were performed by individually classifying each fly as living or dead (see [11] for assessment criteria).

### Data analysis

Mortality data from larval and adult dose-response bioassays were analyzed with probit models using the ecotox package in R 3.3.2 [17, 18]. For larval bioassays, mortality could not be directly measured because the precise number of larvae in each bottle at the time of treatment was not known. We therefore estimated the number of larvae killed by the insecticide treatment (*n*_*k*_) as

$$n_{k}={\bar{n_{c}}-n}_{l},$$

where $\bar{n_{c}}$ represents the average number of adults to eclose from control (i.e., ddH_2_O- or acetone-treated) bottles and *n*_*l*_ is the number to emerge from a given insecticide-treated bottle. In cases where *n*_*l*_ was greater than $\bar{n_{c}},\;n_{l}$ was set to equal $\bar{n_{c}}$.

The proportion of larvae killed per bottle (*P*_*k*_), calculated as

$$P_{k}=n_{k}/\bar{n_{c}},$$

was used as the response variable and was weighted by sample size (i.e. $\bar{n_{c}}$, or the mean number of larvae to survive when no insecticide is present). Concentration (ppm) was log-transformed and included as a predictor variable for each analysis. Separate probit models were used for each insecticide tested. LC_50_ and LC_90_ values as well as their 95% confident limits were calculated.

Adult bioassays using spinosad were also analyzed with probit models, though additional variables for sex (male or female) and strain ID (WAT, WAT-S_5_, WOLF-1, and WOLF-2) were included as well. WOLF-1 and WOLF-2 flies were tested concurrently with WAT and WAT-S_5_ strains, respectively, but otherwise were indistinguishable from each other and came from the same laboratory colony. This approach enabled us to assess sex-specific changes in susceptibility due to selection (WAT vs. WAT-S_5_) while also accounting for any unexplained variation between assessment periods (WOLF-1 vs WOLF-2). These comparisons were made using the ratio_test() function in the ecotox package, which provides a robust method for comparing LC_50_ values from different dose-response curves [19]. Resistance ratios (RRs)were calculated by dividing WAT and WAT-S_5_ LC_50_s by WOLF-1 and WOLF-2 LC_50_s, respectively.

Chi-square analyses were performed in R to compare eclosion rates (i.e. larval susceptibility) from insecticide-treated bottles pre- and post-selection (WAT vs WAT-S_5_; WAT vs WAT-M_5_; WAT-M_5_ vs WAT-M_20_).

## Results and discussion

### Identifying larval lethal concentrations for commonly used insecticides

**Fig 1:**
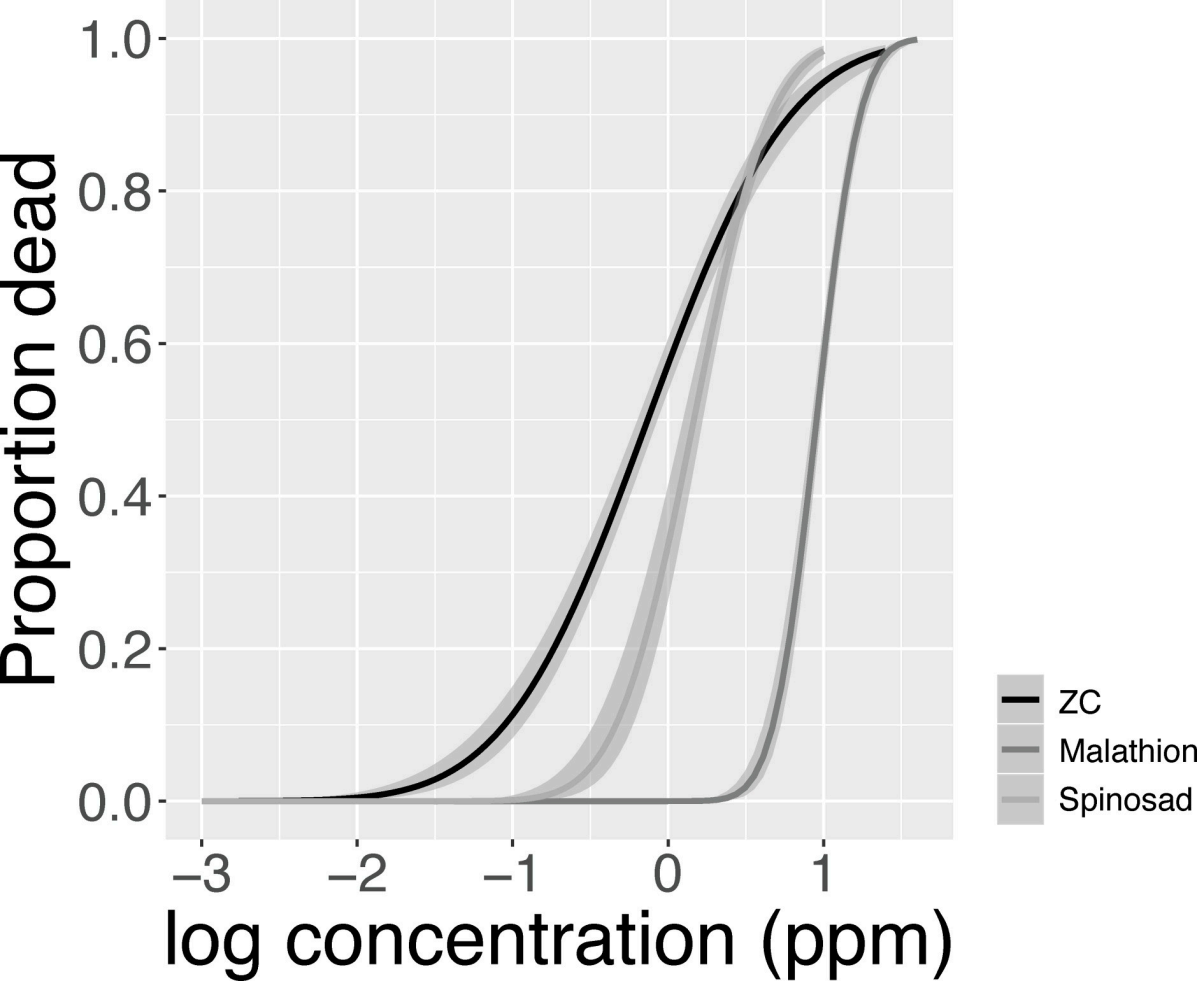
Dose-response relationships for susceptible *D*. *suzukii* larvae exposed to three key insecticides. Mortality was estimated following larval exposure to malathion, spinosad, and zeta-cypermethrin (ZC). Dose-response curves and 95% confidence intervals obtained from probit model.

Data from the larval dose-response analysis, which used susceptible *D*. *suzukii* from the Wolfskill population, show that mortality increased in a log-dose manner, enabling the use of probit models to identify lethal concentrations for each insecticide (Fig 1; S1 Table). LC_50_ and LC_90_ values from these analyses are presented in Table 1. Overall, zeta-cypermethrin exhibited the lowest LC_50_, followed by spinosad and malathion, respectively. However, when comparing LC_90_s, spinosad exhibited the lowest value of the three materials tested. These results differ from previous studies which found that, at the adult stage, spinosad lethal concentration values are greater than both malathion and zeta-cypermethrin [16, 20]. Together, these studies suggest that the relative efficacy of insecticides may differ across life stages or as a result of the different exposure routes they experience (e.g. larvae are surrounded by and feed on fruit containing low concentrations of pesticide). This pattern of larval susceptibility does not appear to be caused by the different solvents used to dilute our insecticides, as the number of flies that emerged from ddH_2_O- ($\bar{x}$ = 58.2, S.E. = 3.28 adults) and acetone-treated ($\bar{x}$ = 59.8, S.E. = 7.32 adults) bottles was similar.

**Table 1:** LC_50_ and LC_90_ values for susceptible *D*. *suzukii* larvae exposed to three commonly used insecticides.

AI	LC_50_ (ppm)	95% CI	LC_90_ (ppm)	95% CI
**Malathion**	9.0	7.6–10.3	17.2	14.9–21.0
**Spinosad**	1.5	0.9–2.0	4.7	3.6–6.8
**Zeta-cypermethrin**	0.7	0.5–1.0	6.2	3.9–12.0

In all cases, larval LC_90_ values fell far below the maximum caneberry field application rate of each material. For malathion, the maximum label rate (2,397 ppm) is 139 times greater than the larval LC_90_ found here; the spinosad label rate (113 ppm) is 24 times higher than the larval LC_90_; and the zeta-cypermethrin label rate (180 ppm) is 29 times higher than the larval LC_90_. However, the concentrations experienced by larvae in the field, within infested fruit, are likely significantly lower than the field application rates. Indeed, a recent study by Andika et al [21] showed that treating *D*. *suzukii* infested cherries with the label rate of various insecticides (including an organophosphate, a pyrethroid, and a spinosyn) significantly reduced, but did not eliminate, larval survival and adult emergence in most instances. For zeta-cypermethrin, specifically, residue concentrations within the fruit were measured across two years, and high variability was observed. In 2018, an average concentration of 3.7 ppm was detected at the subsurface level, whereas 2017 results detected no zeta-cypermethrin residue within the fruit [21]. These findings suggest significant overlap in the range of concentrations tested here and those experienced by *D*. *suzukii* larvae in the field.

### Change in larval susceptibility due to larval selection

During the first generation of larval selection, 8.9% of Watsonville (WAT) *D*. *suzukii*, on average, emerged from bottles treated with the discriminating dose of spinosad (10 ppm), whereas 10.5% emerged from those treated with the discriminating dose of malathion (40 ppm). For both AIs, the discriminating dose used was approximately the LC_90x2_ concentration. In contrast, less than 1% of WOLF *D*. *suzukii* emerged when exposed to the same concentrations, indicating that larvae from the WAT population have experienced notable reductions in susceptibility to these commercially important AIs. After five generations of selection, eclosion rates significantly increased in both lines, reaching 22.2% in WAT-S_5_ (χ^2^ = 40.29, d.f. = 1, p < 0.001) and 30% in WAT-M_5_ (χ^2^ = 62.58, d.f. = 1, p < 0.001). When the malathion line was re-assayed after approximately 20 generations of selection, larval survival increased to 70.6%, representing a significant increase over WAT-M_5_ (χ^2^ = 290.09, d.f. = 1, p < 0.001; Table 2).

**Table 2:** Eclosion success following exposure to discriminating dose before and after selection.

	Average % eclosion (S.E.)		
Selection line	Gen 1	Gen 5	Gen ~20
**spinosad**	8.9(2.1)	22.2(3.5)*	N/A
**malathion**	10.5(2.1)	30.4(2.5)*	70.6(5.4)^+^

* = significantly different from Gen 1 (p < 0.05)

^+^ = significantly different from Gen 5 (p < 0.05)

### Change in adult susceptibility due to larval selection

**Fig 2:**
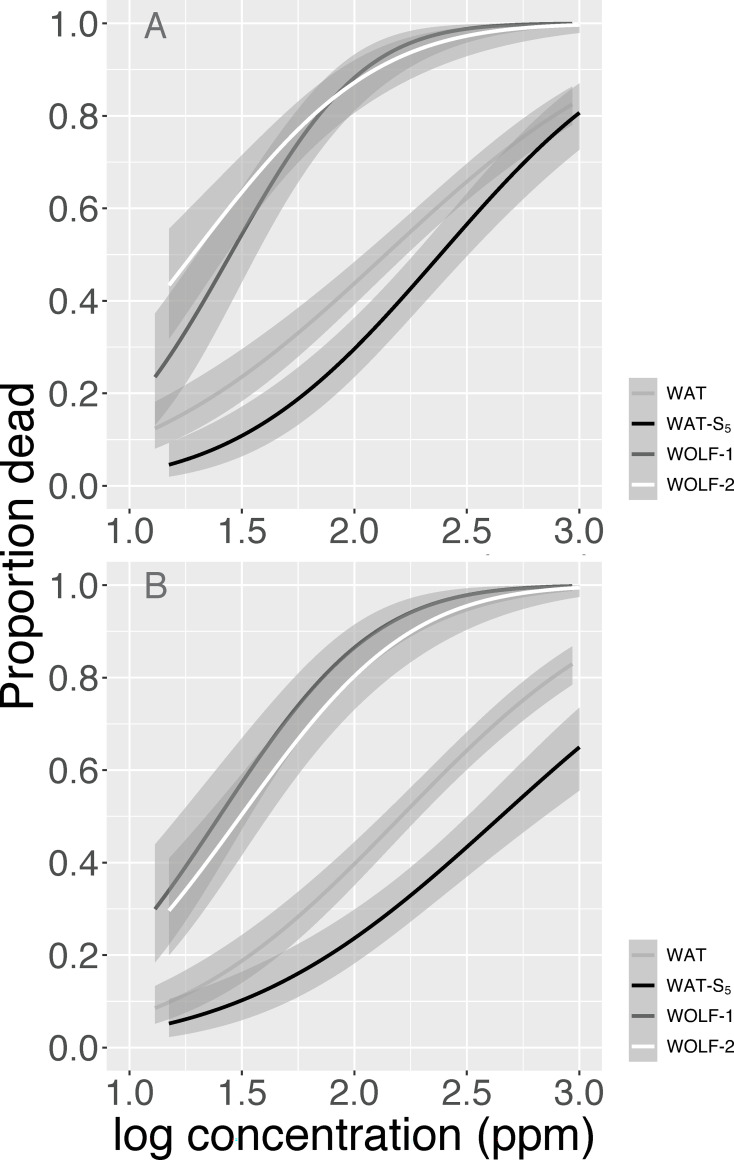
Adult dose-response relationships for *D*. *suzukii* strains. Adult mortality assessed after 8 h exposure to spinosad residues for males (A) and females (B). The Watsonville field population was assessed pre (WAT) and post (WAT-S_5_) five generations of larval selection. Adults from the susceptible Wolfskill strain were simultaneously assayed for comparison during the pre-selection (WOLF-1) and post-selection (WOLF-2) trials. Dose-response curves and 95% confidence intervals obtained from probit models.

The change in adult susceptibility to spinosad due to larval selection was assessed by performing dose-response analysis on Watsonville *D*. *suzukii* before (WAT) and after (WAT-S_5_) imposing five generations of selection. Prior to implementing the larval selection protocol, male and female WAT adults exhibited significantly higher LC_50_ values than their same-sex counterparts from the susceptible Wolfskill population (WOLF-1) (Fig 2; Tables 3 & 4). Specifically, we observed RRs of 4.9 and 6.8 for WAT males and females, respectively (Table 3). These values are similar to those reported by Gress and Zalom [11] which tested the same field populations in fall of 2017 and found RRs of 4.3 for males and 5.2 for females. Following larval selection, WAT-S_5_ males exhibited LC_50_s 75% higher than pre-selection WAT males (Fig 2A), similar to the 86% increase observed using the adult selection method [11]. In contrast, female resistance increased more rapidly with larval selection (177%; Fig 2B) than with adult selection (49%). As a result, WAT-S_5_ RRs increased to 12.6 for males and 14.4 for females when compared to the susceptible strain. It is currently unclear what accounts for the sex-specific response patterns observed in these studies, and future work should investigate this question in greater detail.

**Table 3:** Adult susceptibility of WOLF-1, WOLF-2, WAT and WAT-S_5_ *D*. *suzukii* to spinosad.

Strain	Sex	n	LC_50_ (ppm)	95% CI	RR
**WOLF-1**	male	275	28.1	16.6–40.6	-
	female	275	23.8	11.2–40.6	-
**WAT**	male	685	137.8	99.9–182.5	4.9
	female	685	161.6	124.6–205.0	6.8
**WOLF-2**	male	255	19.1	8.7–30	-
	female	255	31.2	17.3–47.4	-
**WAT-S_5_**	male	295	241.1	160.3–377.6	12.6
	female	295	448.4	279.3–884.3	14.4

**Table 4:** Results of pairwise ratio tests comparing LC_50_s among strains of *D*. *suzukii*.

Comparison	SE	Z	P value
***male***			
**WOLF-1 –WOLF-2**	0.082	2.047	0.041
**WOLF-1 –WAT**	0.05	13.89	< 0.001
**WOLF-2 –WAT-S_5_**	0.073	15.07	< 0.001
**WAT–WAT-S_5_**	0.034	7.208	< 0.001
***female***			
**WOLF-1 –WOLF-2**	0.1	1.175	0.24
**WOLF-1 –WAT**	0.091	9.108	< 0.001
**WOLF-2 –WAT-S_5_**	0.055	21.01	< 0.001
**WAT–WAT-S_5_**	0.037	11.96	< 0.001

When comparing adult mortality in the Wolfskill population during the first (WOLF-1) and second (WOLF-2) assessment periods, we found no significant difference in female susceptibility (Table 4). Males, however, exhibited a marginally significant decline in LC_50_ during the second assessment (Table 4). This finding raises the possibility that laboratory conditions or other sources of error could have increased the potency of spinosad residues during the second assessment period. If true, our results may underestimate the real evolutionary potential for resistance to increase in the near-term. Nevertheless, this study provides the first evidence that larval selection can be used as an effective tool for performing resistance risk assessments in *D*. *suzukii* and is capable of increasing both larval and adult tolerance to the target AI(s). In a previous study, Smirle et al [20] attempted to select for malathion resistance in *D*. *suzukii* from British Columbia, Canada using a different larval selection protocol developed for *D*. *melanogaster*, but the concentration of malathion used had no impact on *D*. *suzukii* larval survival or eclosion success. Perhaps unsurprisingly, no malathion resistance was observed despite 30 generations of exposure [20]. By identifying lethal concentrations of commonly used insecticides for susceptible *D*. *suzukii* larvae, this study provides a valuable resource for researchers looking to perform resistance monitoring or risk assessments on field populations of interest. We expect that this information will become increasingly more important as the widespread use of these critical insecticides results in the loss of susceptibility throughout North America, South America and Europe. To this point, a recent study by Mishra et al [22] found that *D*. *suzukii* collected from commercial blueberry fields in Georgia exhibited a 3-fold increase in tolerance to malathion, spinosad, and zeta-cypermethrin relative to flies from an untreated location, indicating that resistance could soon emerge in other locations.

The larval bioassay, whether used for resistance monitoring or selection studies, also offers several advantages over the adult glass vial approach that makes it appealing across many experimental contexts. First, implementing this protocol requires little time, effort or cost investment beyond what is already required for standard *D*. *suzukii* colony maintenance. In contrast, the glass vial approach used for adults requires 1) purchasing large numbers of glass scintillation vials, 2) rolling treated vials throughout the drying process to help ensure residues are evenly distributed, and 3) manually loading adult flies into each vial to perform the bioassay. The loading process can be performed either using CO_2_ to anaesthetize the flies or by gently aspirating the adults into the vial, and both methods have the potential to harm or kill the flies in the process. Additionally, 4) adult mortality assessments can be time-consuming and require subjective assessments of how to classify individuals at varying stages of moribundity. With larval bioassays, survival can easily and objectively be estimated by counting the number of adults to emerge from treated and untreated bottles.

Finally, because each adult female is capable of laying multiple eggs per day, a fraction of the number of adult *D*. *suzukii* are needed to conduct resistance monitoring and risk assessment studies with the larval protocol. For example, using the adult selection method adopted by Gress and Zalom [11] if a group of 100 female *D*. *suzukii* were exposed to their LC_90_ concentration, on average, 10 survivor females would remain to produce the subsequent generation. In contrast, if the same 100 females were split among 5 bottles (n = 20 females per bottle) and the larval selection method was implemented using the LC_90_, our results indicate that, on average, more than 30 survivor adults (> 50% of which would be female) would emerge. Moreover, because the larval bioassay does not require killing the parental females, these flies can continue to produce new progeny, thus drastically expanding the number of potential offspring. Although we did not measure the extent to which oviposition rate or larval susceptibility changes with maternal age, previous work has shown that female reproductive output remains high for up to 90 days post-eclosion when maintained on artificial cornmeal diet [23]. Together, these findings indicate that larval bioassays are a simple, cost-effective approach for measuring the current state of insecticide resistance in field populations as well as the potential for greater resistance to develop. Greater effort to monitor these trends in *D*. *suzukii* field populations is needed to ensure that appropriate resistance management actions can be implemented when early susceptibility loss is detected.

## Supporting information

S1 Table Data for the larval dose-response bioassay study performed in the subsection titled “identifying larval lethal concentrations of commonly used insecticides”. (CSV)

S2 Table Adult emergence data for the experiment performed in the “larval selection and susceptibility” subsection. (XLSX)

S3 Table Data for the adult bioassay study performed in the subsection titled “impact of larval selection on adult susceptibility”. (CSV)
